# Prolactinomas in Moroccan Population: Clinical, Paraclinical, Therapeutic, and Evolutionary Aspects

**DOI:** 10.7759/cureus.31547

**Published:** 2022-11-15

**Authors:** Zineb Elazime, Mohammed Amine Essafi, Nourelhouda Remok, Hayat Aynaou, Houda Salhi, Hanan El Ouahabi

**Affiliations:** 1 Department of Endocrinology, Diabetology, Metabolic Diseases and Nutrition, Hassan II University Hospital, Fez, MAR; 2 Faculty of Medicine and Pharmacy, Sidi Mohamed Ben Abdellah University, Fez, MAR

**Keywords:** tumors, dopamine agonists, hyperprolactinemia, pituitary adenoma, prolactinoma

## Abstract

Prolactinomas are usually benign tumors and are the most common type of secretory adenomas. Their diagnosis is well coded, and their severity depends on the tumor size identified by magnetic resonance imaging.

The aim of this work is to study the epidemiological, clinical, paraclinical, and therapeutic profile of prolactinomas through a retrospective and descriptive study of 95 cases of prolactinomas conducted at the Endocrinology, Diabetology and Nutrition Department of the Hassan II University Hospital of Fez between January 2015 and November 2020.

The mean age of patients in our series was 33.8 years (14-63) with a clear female predominance of 88.4%. Based on adenoma size, 37 cases were microprolactinomas and 58 cases were macroprolactinomas representing 38.9% and 61.1% respectively. Menstrual disorders were present in 50.5% of the women, and 68.4% had galactorrhea, while 44.2% of patients complained about local mass effects, especially headaches. The average size of adenomas was 15.7 mm (4-70 mm). The mean initial plasma prolactin (PRL) level was 423.2 ng/mL (103-7,663 ng/mL). Of our patients, 97.9% received medical therapy with dopamine agonists, particularly with cabergoline which represents the mainstay of therapy (85.3%), while 2.1% of patients underwent surgery due to resistance to treatment.

In terms of evolution after treatment, three cases of resistance to treatment were reported. Thirty patients with microprolactinomas had a normalization of prolactin level within an average of five months, and we saw a regression of the size of the adenoma in 43.2% of patients, stabilization in 24.3% and a progression of the process in 5.4% of cases. Although in macroprolactinomas we found a normalization of prolactin level in 43 patients within an average of one year, the tumor syndrome disappeared in 31.6% of cases. Radiological monitoring also revealed a favorable tumor response in 82.7% of patients.

## Introduction

Prolactinomas are the most common types of pituitary adenomas [1], with an average age at discovery between 20 and 50 years. They are most often sporadic and about 5% of cases are parts of genetic syndromes [2]. The diagnosis of prolactinomas is based on the quantitative determination of the prolactin level in the blood as well as on magnetic resonance imaging to evaluate the size of the tumor. Their management has been transformed by the use of dopamine agonists (DAs) that were introduced in the 70s.

This study aims at investigating the epidemiological, diagnostic, therapeutic and evolutionary aspects of a series of 95 cases of prolactinomas followed in the Department of Endocrinology, Diabetology and Nutrition of the Hassan II University Hospital over a period of five years.

## Materials and methods

Study and population

A retrospective, descriptive and analytic study was conducted over a period of five years. The study reviewed patients with prolactinoma who were hospitalized in the Endocrinology Diabetology and Nutrition Department of the Hassan II Hospital in Fez.

Patients whose medical records could be used for the various clinical, paraclinical, therapeutic, and evolutionary aspects of prolactinomas were included, while those with incomplete medical records, in whom hyperprolactinemia was not attributable to a secretory pituitary adenoma, or lost to follow-up, were excluded from the study.

Data collection

Data were collected from the patients' medical records using a pre-established data-exploitation file and were reported on an exploitation form, then integrated into a computer database. The sociodemographic variables were age and gender. The clinical variables were medical history related to hyperprolactinemia and clinical examination including general examination of breast and external genital organs. Paraclinical variables were baseline serum prolactin level, pituitary axis hormones evaluation, hypothalamic-pituitary MRI, ophthalmologic evaluation with the fundoscopic exam, visual field testing, transthoracic echocardiogram (TTE), and other explorations. Evolutionary aspects were identified based on clinical features, prolactin serum measurement, and hypothalamic-pituitary MRI.

Statistical analysis

Both qualitative and quantitative (means and standard deviations) variables were measured in this study. We used the classical parametric test (chi2 test) to test associations between categorical variables. In all the analyses, we kept the level of significance at a p-value lower than 0.05. We performed the statistical analysis using SPSS software (IBM Corp. Released 2015. IBM SPSS Statistics for Windows, Version 23.0. Armonk, NY: IBM Corp).

Ethics statement

Participants' anonymity and confidentially was highly respected.

## Results

The sociodemographic features

The study reviewed 95 patients with a female predominance (88.4%), with a sex ratio of 0.13. As for the age variable, the average age of diagnosis of prolactinoma was 33.8 years, with a higher incidence (32.6%) among the 31-40 age group.

Clinical and biological characteristics

Three patients reported head trauma (3.2%), and none had received cranial radiotherapy. The gynecological history reported by 84 women in our study included irregular menstrual cycle in 48 patients (50.5%), infertility in 10 patients (10.5%), and menopause in nine patients (9.5%). Five patients reported the use of drugs (5.3%). Only one patient reported a similar family case (1.1%). Multiple endocrine neoplasia type 1 was found in one patient associating adrenal hyperplasia with primary hyperparathyroidism and prolactinoma (Table 1).

**Table 1:** Functional and Clinical Features Reported According to Sex and Age

Functional and clinical and biological features		Cases	Percentage %
Female	Spontaneous galactorrhoea	35	36.8%
	Induced galactorrhoea	65	68.4%
	Spaniomenorrhea	20	21.1%
	Amenorrhea	28	29.5%
	Infertility	10	10.5%
	Menopause	9	5.3%
	Vaginal dryness	1	1.1%
	Dyspareunia	1	1.1%
Male	Gynecomastia	4	4.2%
	Regression of secondary sexual features	2	2.1%
Child	Early-onset puberty	1	1.1%
Both sexes	Headache	42	44.2%
	Decreased visual acuity	35	36.8%
	Head Trauma	3	3.2%
	Decrease in libido	6	6.3%
	Similar family case	1	1.1%
	History of MEN1	1	1.1%
Prolactin baseline	100–150ng/ml	25	26.3%
	150–200 ng/ml	40	42.1%
	>200 ng/ml	30	31.6%

With women, breast examination revealed induced galactorrhoea in 65 patients (68.4%), and spontaneous galactorrhoea in 35 patients (36.8%), while with men, gynecomastia was found in four patients (4.2%), and regression of secondary sexual features was reported in two patients. We performed a serum prolactin measurement in all patients (Table 1).

Radiological features

**Figure 1:**
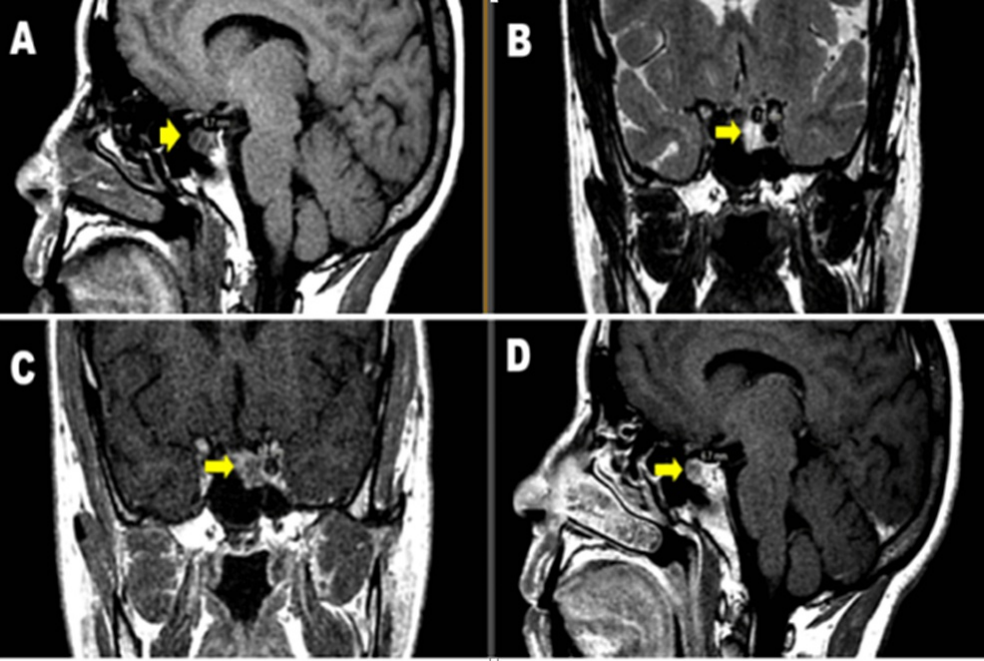
MRI images: sagittal T1 (A), coronal T2 (B), coronal T1 C + (C), and sagittal T1 C + (D) showing a 7-mm anteropituitary microadenoma in hyposignal T1, hypersignal T2 (Department of Radiology CHU Hassan II Fez).

**Figure 2:**
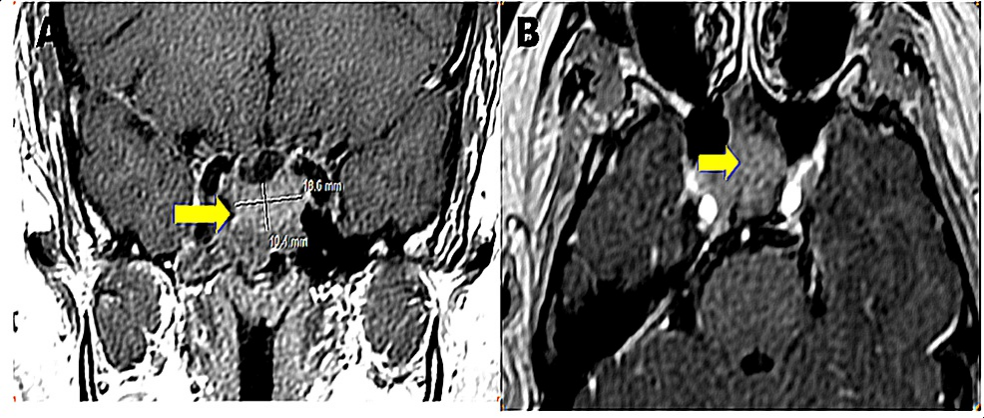
Injected MRI images: coronal T1 (A) and axial T2 (B) showing a pituitary macroadenoma invading the cavernous sinus and enveloping the right internal carotid artery. It also invades the sellar bottom and the large wing of the right sphenoid (Department of Radiology CHU Hassan II Fez).

A hypothalamic-pituitary MRI was performed on all patients (Figures 1A-1D, 2A, 2B).

Side effects of prolactinomas

Pituitary Axis Hormones Evaluation

Hypogonadotropic hypogonadism was found in 58.9% of the population, corticotropic insufficiency in 15.8%, and hypercorticism in one patient, reflecting a mixed secretion of adrenocorticotropic hormone (ACTH) and prolactin (PRL). Central hypothyroidism was found in 21 patients (22.1%), while one patient had a mixed PRL/TSH secretion. An elevated level of IGF-1 was reported in five patients (5.3%) who presented an absence of GH suppression on the oral glucose tolerance test, confirming the diagnosis of a mixed PRL/GH adenoma. No cases of diabetes insipidus were reported.

Fundoscopic and Visual Field Results

We performed a fundoscopic examination of 58 patients (61.1%) with macroprolactinoma and evaluated the visual field in all patients. Table 2 demonstrates the different abnormalities.

**Table 2:** Fundoscopic examination and visual field testing in our series

Evaluations	Results	Cases	Percentage
Confrontation visual field-testing anomalies	Bitemporal hemianopsia	6	6.3%
	Right homonymous hemianopsia	3	3.2%
	Left temporal hemianopsia	2	2.1%
	Right temporal hemianopsia	2	2.1%
	Confrontation visual field testing normal	82	86.3%
Fundoscopic examination	Unspecified abnormality	7	7.4%
	Retrobulbar optic neuropathy	2	2.2%
	*Papillary* pallor	9	9.5%
	*Papillary* edema	5	5.3%
	Normal	35	36.8%

Treatment

We treated 97.9% of patients with dopaminergic agonists, using two molecules, namely cabergoline, and bromocriptine. Cabergoline was initiated at a dose of 0.5 mg/week (½ cp/week) with a progressive increase. Bromocriptine was initiated at a dose of 1.25 mg/d (½ cp/d) with an increase of 1.25-2.5 every 48 hours to achieve therapeutic goals with a maximum dose of 10mg/d. Before treatment, TTE revealed minimal valvulopathy in seven patients who were referred to the cardiology department for regular follow-up after initiating cabergoline therapy. The indication of surgery was established in 2.1% of the cases for mixed adenomas, one with PRL/TSH and the others with PRL/GH.

During the follow-up, the indication of surgery was given to three other patients due to their resistance to dopaminergic agonist treatment.

Evolutionary aspects

Microprolactinoma

**Figure 3:**
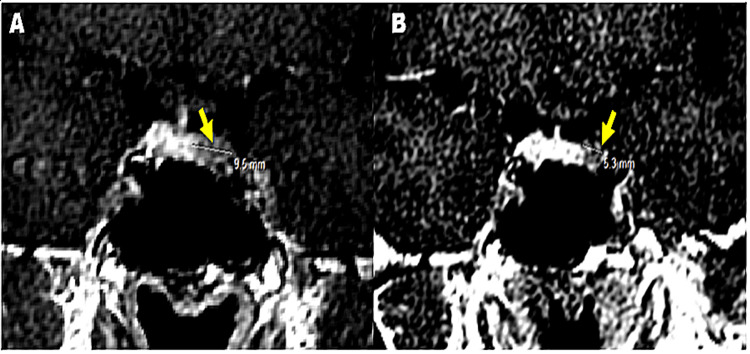
T1 C + coronal injected MRI images before (A) and after treatment T2 (B): decrease in size of the adenoma measuring 5 vs 10 mm (Department of Radiology CHU Hassan II Fez).

We followed 37 patients for a mean of 3.5 years. A return to regular menstrual cycles was noticed in 14 patients (14.7%) with the disappearance of induced galactorrhea in 20 patients (21%). Thirty patients had a normalization of prolactin within an average of five months. We performed a control hypothalamic-pituitary MRI in 30 patients after one year, which revealed a regression of the size of the adenoma in 16 patients (43.2%), the disappearance of the microprolactinoma in three patients (8.1%), a stabilization in nine patients (24.3%) and an aggravation of the process in two patients (5.4%) (Figures 3A, 3B).

Macroprolactinoma

We followed 58 patients for a mean duration of 4.32 years. A return to regular menstrual cycles was noted in 28 patients (29.5%), the disappearance of galactorrhea in 35 patients (36.8%), and the disappearance of the tumor syndrome in 30 patients (31.6%). As for the normalization of prolactin level, it was detected in 43 patients within an average of one year. Additionally, radiological monitoring by MRI performed at three months of treatment and then annually for five years revealed a favorable tumor response in 82.7% of patients.

Dopamine Agonist-Resistant Macrolactinoma

**Figure 4:**
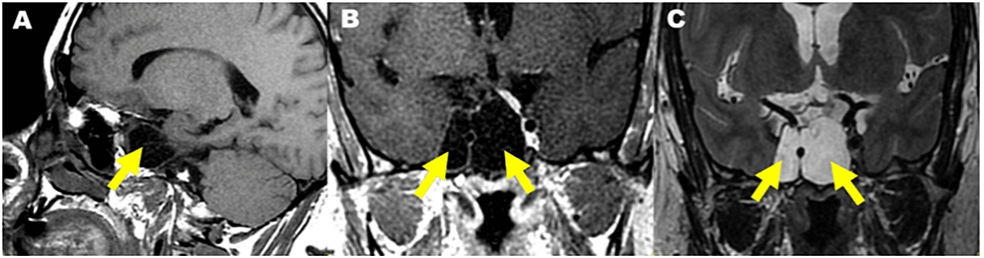
Injected MRI images: sagittal T1 (A), coronal T1 (B), coronal T2 (C): showing a cystic pituitary macroadenoma with a liquid component in T1 hyposignal, T2 hypersignal, without enhancement (Department of Radiology Hassan II University Hospital, Fez).

We noted three cases of resistance to treatment. The first case was a 38-year-old patient without any notable pathological history, who was followed up for a 70-mm macroprolactinoma with signs of invasion on MRI and was put on cabergoline at maximum dose stepwise (3.5 mg/week). During the follow-up, the control MRI showed cystization of the adenoma, which increased in size. The patient was then put on bromocriptine and in light of the resistance, she was referred to the neurosurgery department for further management. MRI images of this patient are shown in Figures 4A-4C.

We followed the other two patients for invasive macroadenomas larger than 4 cm on cabergoline at a maximum dose of 4.5 mg/week without normalization of PRL or regression of tumor size on MRI. The management of these patients consisted of the substitution of cabergoline for bromocriptine and the indication of surgical treatment. Considering the young age and the pregnancy project of the first patient, she underwent an almost complete surgical removal of her adenoma. The postoperative follow-up was marked by the normalization of PRL, the disappearance of the adenoma, and the return of menstrual cycles. The second patient is still waiting for surgery.

 *Evolution of the Other Endocrine Axis*

After the normalization of PRL, normalization of the gonadotropic axis occurred in 67.4% of the population with a mean time of 8.6 months. During the follow-up, four patients developed a corticotropic insufficiency, three of them after a pituitary apoplexy and the last after an increase of the tumor volume, requiring a hormonal replacement treatment with hydrocortisone and L-thyroxin.

## Discussion

Prolactinomas represent the most common type of pituitary adenomas [3,4], and the average age of discovery is 30 with a female predominance [1]. It is typically manifested by amenorrhea-galactorrhea syndrome with a serum prolactin level higher than 20 ng/mL in men and 25 ng/mL in women [5].

Magnetic resonance imaging (MRI) can reveal tumor lesions of variable size and extensions [6]. In all series, the signs of invasion concern macroprolactinomas, and no cases of invasive microprolactinomas were reported. Indeed, according to Francey et al. [7], gonadal insufficiency is the most frequently compromised axis in patients with prolactinoma, with 69.8%. Other axes can also be compromised, respectively, the thyroid axis (26.2%) followed by the corticotropic axis (5%). In fact, the order of frequency was similar to the cases of our patients.

Francey et al. found a mixed PRL/GH adenoma in five patients, while only one patient had a mixed PRL/TSH adenoma [7]. Actually, in our series, we found hormonal co-secretion in the same hormones and the same order of frequency since six patients presented mixed adenomas: one with PRL/TSH and the others with PRL/GH.

In terms of treatment, not all pituitary adenomas are necessarily treated. The decision to treat depends on the size of the tumor and the impact on the gonadotropic axis [8]. The treatment is mainly based on the class of dopaminergic agonists since it allows the control levels of prolactin after four to six weeks [9]. Because of their safety and efficacy, cabergoline and quinagolide are used as first-line drugs [7]. In fact, according to the series of Verhelst et al. [10], Francey et al. [7], of Cannavo et al. [11] as well as our study, cabergoline is the dopaminergic agonist of choice in the treatment of prolactinomas. The second molecule used in both the Verhelst et al. series and ours is bromocriptine. According to Colao et al. [12], the initial average dose of cabergoline varies between 0.5-1mg/Sem, while the dose of bromocriptine varies between 2.5 and 15 mg/d. Thereafter, the dosage used with our patients is close to that approved in the literature, with a mean initial dose of cabergoline between 0.25 and 0.5 mg/week (i.e., ½-1cp/Sem), and between 1.25-10 mg/d of bromocriptine (0.5-4 cp/d).

The study by Kars et al. [13] highlighted a statistically significant increase in the prevalence of discrete (asymptomatic) tricuspid insufficiency in patients on cabergoline compared to the control group with no significant difference in the prevalence of moderate or severe tricuspid insufficiency. Also, an increased risk of heart valve failure has been reported in patients treated with certain dopaminergic agonists for Parkinson's disease [14]. Before starting cabergoline, it is necessary to perform transthoracic echocardiography of the heart and then regularly throughout the treatment, if a patient is taking a total weekly dose of more than 2 mg, then annual echocardiography is recommended [15]. There is also literature that recommends monitoring, especially for doses higher than 3 mg per week and/or in the long term [16]. In our series, TTE revealed minimal valvulopathy in seven patients who were referred to the cardiology department for regular follow-up after initiating cabergoline therapy.

According to a study conducted in 2003 by Colao et al. [15], which evaluated the risk of recurrence of hyperprolactinemia in patients treated with cabergoline, the rate of recurrence was only 30% for microprolactinomas and 36% for macroprolactinomas over a period of 12-18-month follow-up. These encouraging results led to the determination of criteria for discontinuation of treatment, which are summarized as normal prolactin under a low dose of cabergoline (0.5 mg/week) and disappearance or 50% decrease in tumor size on MRI. In our series, we stopped medical treatment with 18 patients meeting these criteria without any evidence of tumor recurrence during the follow-up.

In addition, surgery is not justified in the first line. The surgical technique is an extended exeresis of the adenoma by a transsphenoidal rhinoseptal approach through a transnasal or sub-labial approach. In fact, the sub-labial approach has been abandoned and replaced by the transnasal one, which is considered faster and simple to perform, less hemorrhagic, and finally less traumatic [17]. Besides, by adopting this approach, we can expect 75% to 90% normalization of prolactin levels in the immediate postoperative period and restored fertility in more than 80% of cases. However, the recurrence rate at five years is estimated at 20%. Currently, intracranial irradiation includes stereotactic radiosurgery (RS) and fractionated intracranial stereotactic radiotherapy (FIRST) [18]. The side effects described for this type of treatment include panhypopituitarism, optic neuropathy, cerebral vascular accident, and secondary tumors [19]. This treatment is almost never used nowadays because of the effectiveness of medical and surgical treatments in the vast majority of cases, also, because of its adverse effects and the long duration, it takes for the PRL to normalize, both for RS and FIRST [20].

In terms of clinical evolution after treatment, the biological evolution in the series of Verhelst et al. [10] noticed a normalization of PRL in 84% of patients, 92% of microprolactinomas, and 77% of macroprolactinomas, whereas in the series of Colao et al. [12] the normalization of prolactin level concerned 61% of microprolactinomas and 47% of macroprolactinomas. In essence, the biological evolution in our study was favorable in 81% of microprolactinomas and 74.1% of macroprolactinomas. Radiologically, according to Verhelst et al. [10], a favorable tumor evolution was noted in 67% of the patients compared to 80% (76 patients divided into 28 microadenomas and 48 macroadenomas) in our series.

The study by Vroonen et al. on a group of 92 prolactinomas defined resistance as an inability to control prolactin levels at a maximum dose of 2 mg per week for a minimum of six months [21]. Resistance seems to be mainly in invasive macroadenomas (>80% of cases) and also in the male gender and prolactin levels are higher at diagnosis. In our series, three cases of resistance to cabergoline were reported. All three were invasive macroprolactinomas larger than 4 cm, the decision was to substitute the cabergoline with bromocriptine and to refer them to the Neurosurgery department.

Limitations and strengths of the study

Although the sample size is interesting, since it allowed us to study several parameters as well as to appreciate the evolutionary aspects of prolactinomas, it is still less representative of the general population in the country which makes it difficult to generalize the results.

## Conclusions

Our study highlights the prevalence of prolactinomas in the Moroccan population and the main clinical and paraclinical features that could help physicians diagnose and provide earlier health care. The favorable evolution of medical therapy, instaured after precautionary measures, especially cardiac, facilitates the management of prolactinomas and reduces invasiveness in terms of treatment.

Long-term monitoring should be planned to adjust the therapeutic doses according to the prolactin level, to detect complications related to the increase in tumor size or pituitary apoplexy, and to better understand the problems induced by estrogens in case of pregnancy or contraception. However, there are several controversies mainly about the rare cases of resistance to dopaminergic agonists or prolactin carcinoma. Similarly, there is no clear consensus on the decision to discontinue therapy if the disease is controlled.
